# Luminescent multiplex viability assay for *Trypanosoma brucei gambiense*

**DOI:** 10.1186/1756-3305-6-207

**Published:** 2013-07-15

**Authors:** Nick Van Reet, Pati Pyana, Stijn Rogé, Filip Claes, Philippe Büscher

**Affiliations:** 1 Department of Biomedical Sciences, Institute of Tropical Medicine, Nationalestraat 155 2000 Antwerp, Belgium; 2 Institut National de Recherche Biomédicale, Avenue de la Démocratie, BP 1179, Kinshasa-Gombe, Democratic Republic of the Congo

**Keywords:** *Trypanosoma brucei gambiense*, Democratic Republic of the Congo, Viability, Multiplex, Luminescence, *Renilla* luciferase

## Abstract

**Background:**

New compounds for the treatment of human African trypanosomiasis (HAT) are urgently required. *Trypanosoma brucei* (*T.b.) gambiense* is the leading cause of HAT, yet *T.b. gambiense* is often not the prime target organism in drug discovery. This may be attributed to the difficulties in handling this subspecies and the lack of an efficient viability assay to monitor drug efficacy.

**Methods:**

In this study, a *T.b. gambiense* strain, recently isolated in the D.R. Congo, was made bioluminescent by transfection with *Renilla* luciferase (*RLuc*) without altering its *in vitro* and *in vivo* growth characteristics. A luminescent multiplex viability assay (LMVA), based on measurement of the *Renilla* luciferase activity and the ATP content of the cells within the same experiment, was investigated as an alternative to the standard fluorimetric resazurin viability assay for drug sensitivity testing of *T.b. gambiense*.

**Results:**

In a 96-well format, the RLuc transfected strain showed a detection limit of 2 × 10^4^ cells ml^-1^ for the *Renilla* luciferase measurement and 5 × 10^3^ cells ml^-1^ for the ATP measurement. Both assays of the LMVA showed linearity up to 10^6^ cells ml^-1^ and correlated well with the cell density during exponential growth of the long slender bloodstream forms. The LMVA was compared to the fluorimetric resazurin viability assay for drug sensitivity testing of pentamidine, eflornithine, nifurtimox and melarsoprol with both the wild type and the RLuc transfected population. For each drug, the IC_50_ value of the RLuc population was similar to that of the wild type when determined with either the fluorimetric resazurin method or the LMVA. For eflornithine, nifurtimox and melarsoprol we found no difference between the IC_50_ values in both viability assays. In contrast, the IC_50_ value of pentamidine was higher when determined with the fluorimetric resazurin method than in both assays of the LMVA.

**Conclusions:**

LMVA has some advantages for viability measurement of *T.b. gambiense*: it requires less incubation time for viability detection than the fluorimetric resazurin assay and in LMVA, two sensitive and independent viability assays are performed in the same experiment.

## Background

Human African trypanosomiasis (HAT), or sleeping sickness, is caused by two subspecies of *Trypanosoma brucei (T.b.)* and is transmitted through tsetse flies (*Glossina spp*). *T.b. rhodesiense* causes an acute form of sleeping sickness in East Africa. *T.b. gambiense* is responsible for the chronic form in West and Central Africa and accounts for more than 95% of the near 10,000 sleeping sickness patients that are diagnosed and treated annually [1]. In both forms, the disease evolves from a first stage with peripheral tissue invasion, towards a second stage with invasion of the central nervous system. The drugs for treating sleeping sickness are subspecies specific due to their different metabolisation, and are disease stage specific depending on their ability to cross the blood–brain-barrier [2]. *T.b. gambiense* HAT is treated with pentamidine (first stage) and nifurtimox-eflornithine combination therapy or melarsoprol (second stage). *T.b. rhodesiense* HAT is treated with suramin (first stage) or melarsoprol (second stage). All these drugs are toxic and require intramuscular or intravenous injection except for nifurtimox which is an oral drug [3]. Research into new drugs for HAT aims at drugs that are safe, that are active against both subspecies and both disease stages, that can be given orally and that need only one administration [4]. Whole cell *in vitro* high-throughput screenings (HTS) are now in use to discover novel trypanotoxic compounds. However, these HTS assays are almost exclusively performed with one particular non human pathogenic strain: *T. b. brucei* strain *427*[5-14]. Less often a hit is confirmed *in vitro* and *in vivo* on a collection of *Trypanozoon* strains, including *T.b. rhodesiense* and *T.b. gambiense*[15-20]. To be relevant for the current situation in the field, *T.b. rhodesiense* and *T.b. gambiense* strains that are recently isolated from patients with known treatment outcomes and that underwent few *in vivo* and *in vitro* passages, should be included in these drug discovery validation panels [21-23]. It would be even better to include *T.b. gambiense* strains already in the initial HTS screening, because, despite the high sequence similarity between the genomes of *T.b. brucei* and *T.b. gambiense*[24], the latter is often found to be more susceptible to drugs than other *T. brucei* subspecies, as is the case for eflornithine and pentamidine [15,16,25-27]. Several factors hamper inclusion of *T.b. gambiense* as a primary target organism in HTS. *T.b. gambiense* is particularly difficult to isolate from patients and to adapt to mice and to *in vitro* culture [23,28]. Often, *T.b. gambiense* leads to silent or chronic infections in mice with hardly detectable parasites [29]. Generally, bloodstream form parasites from *in vivo* or *in vitro* cultures are exposed to compounds for up to 72 hours, whereafter the remaining viability of the cells is assessed using either radioactive, colorimetric, fluorimetric, or luminescent detection [5,9,26,30,31]. The fluorimetric resazurin viability assay is very cost-effective, but performance is limited with *T.b. gambiense* strains due to lengthy incubation times with resazurin before detection of resorufin yields high enough signal to background for detection. The reason for low conversion of resazurin to resorufin is unknown, but long term incubation times with resazurin with live or lysed trypanosomes may affect the IC_50_ value of a drug [26,31,32]. Because of their easy, sensitive and fast readout, viability assays based on ATP detection (such as the luminescent CellTiter-Glo assay) have been proposed and used as an alternative viability assay for HTS in *T.b. brucei* strain 427 [5,7]. Currently, there is no reporter gene based *in vitro* assay employed for HTS compound screening, either for *T.b. gambiense* or for *T.b. brucei* and *T.b. rhodesiense*, unlike for other protozoan parasites such as *Plasmodium falciparum, Leishmania spp.* and *Trypanosoma cruzi*[33-39]. *Renilla* luciferase tagged parasites have previously been generated for *T.b. brucei* and *T.b. gambiense* and have proved effective for *in vivo* parasite tracking in murine models of experimental trypanosomiasis [29,40] and for preclinical *in vivo* drug efficacy testing [41]. Yet up to now, no *in vitro* application of *Renilla* luciferase parasites has been described. Recently, it has been shown that the EnduRen assay, for measurement of vital *in vitro Renilla* luciferase activity, can be combined with the CellTiter-Glo assay as an efficient luminescent multiplex viability assay for HTS compound screening against Dengue [42]. Assays that measure multiple fitness parameters within the same wells, such as multiplex assays, may decrease false-positive rates and increase confidence for hit selection in HTS [43]. In the present study, a *T.b. gambiense* strain that was recently isolated in the Democratic Republic of the Congo was made bioluminescent by expression of *Renilla* luciferase. With this strain, the feasibility of the Enduren/CellTiter-Glo luminescent multiplex viability assay, abbreviated as LMVA, was compared to the fluorimetric resazurin assay for drug sensitivity testing of the main drugs to treat *T.b. gambiense* sleeping sickness: pentamidine, eflornithine nifurtimox and melarsoprol.

## Methods

### Culture media

Iscove’s modified Dulbecco’s medium powder (IMDM) and fetal calf serum (FCS; heat-inactivated; EU approved South American origin) were purchased from Invitrogen. Methylcellulose (5140 mPa.s) was purchased from Fluka. All other culture media ingredients were from Sigma–Aldrich. An HMI-9 based stock solution [44] was adapted to prepare two culture media for use with *in vitro* culture of bloodstream form *T.b. gambiense*[28]. Briefly, HMI-human serum (HH) contains HMI-9 with 15% v/v FCS and 5% v/v heat-inactivated human serum. HMI – human serum – methylcellulose (HHM) is HH containing a final concentration of 1.1% w/v methylcellulose. For fluorescent and luminescent activity assays, HH was prepared from IMDM without phenol red (Invitrogen).

### *T. b. gambiense* MHOM/CD/INRB/2006/23A

*T. b. gambiense* strain MHOM/CD/INRB/2006/23A, alias 348 BT, was isolated in Mbuyi-Mayi in the Democratic Republic of the Congo in 2006, from the blood of a second stage patient who was cured after a 10 day melarsoprol treatment [45]. The isolation of the bloodstream form *in vivo* in rodents and the adaptation *in vitro* to HHM and the confirmation of its *gambiense* type I genotype have been described previously [23,28].

### *In vitro T.b. gambiense* culture

An HHM adapted population of 348 BT, was inoculated in 500 μl of HHM in a 48-well plate at densities between 10^3^ – 10^5^ cells ml^-1^ and maintained in logarithmic phase growth by sub passages at appropriate dilutions after 24 to 72 hours of incubation at 37°C and 5% CO_2_. Cultures were monitored by phase contrast inverted microscopy. For cell counting, 20 μl were withdrawn and dispensed in a disposable Uriglass counting chamber (Menarini). Cultures were stepwise scaled up to 40 ml, by addition of four volumes of fresh medium in 25 cm^2^ flasks once the parasites reached a density of 5 × 10^5^ cells ml^-1^. For long term storage, cells were cryostabilised in liquid nitrogen as tenfold concentrated log phase cultures in 90% HH with 10% glycerol.

### *In vivo T.b. gambiense* culture

All animal experiments were approved by the Animal Ethics Committee of the Institute of Tropical Medicine Antwerp, under licence PAR019. Female OF-1 mice (40 – 50 g) (Charles River, Belgium), either immune suppressed with 200 mg kg^-1^ cyclophosphamide (Endoxan, Baxter) 48 h before infection or not, were infected intraperitoneally with 2 × 10^5^ parasites in 200 μl, obtained from rodent blood or *in vitro* culture, and diluted at least 1:1 in phosphate buffered saline + 1% glucose pH 8.0 (PSG) [46]. The matching method was used to monitor parasitemia in tail-blood [47] for 4 weeks after which the animals were sacrificed.

### *Renilla* luciferase *T.b. gambiense*

The pHD309 RLuc expression vector, containing *RLuc* cDNA from pGL4 vector (Promega) was used for transfection [40]. Parasites from flask cultures were harvested at 5 × 10^5^ cells ml^-1^ and washed twice in cytomix (2 mM EGTA, 120 mM KCl, 0.15 mM CaCl_2_, 10 mM potassium phosphate pH 7.6, 25 mM Hepes, 1 mM hypoxanthine, 5 mM MgCl_2_, 5 g l^-1^ glucose, 100 mg l^-1^ BSA). Next, they were concentrated to 5 × 10^7^ cells ml^-1^ and 400 μl of this suspension was transferred into a 4 mm cuvette (BioRad), whereafter 50 μl containing 10 μg of *Not*I (New England Biolabs) linearised pHD309 RLuc DNA was added. Subsequently, this mixture was pulsed once in a Gene Pulse Xcell electroporator (BioRad; 1250 V, 25 Ω, 50 μF), transferred to 12 ml of HH, plated in a 48-well plate in 250 μl volumes and incubated at 37°C for 24 h. Next, 250 μl of HH containing 2 μg ml^-1^ hygromycin was added. Populations were maintained in 1 μg ml^-1^ hygromycin for four weeks before cryostabilisation. The *Renilla* luciferase assay system (RLAS, Promega) was used to measure the RLuc activity from lysed cells. Forty μl of trypanosome suspension was mixed with 10 μl of 5 x *Renilla* lysis buffer and 20 μl of this solution was mixed with 100 μl of RLAS assay reagent (via dispenser) in a white opaque 96-well plate (Perkin Elmer). Each time an aliquot was dispensed into a well, the plate was shaken for 2 seconds and after a further 2 seconds delay, the number of photons per second (CPS) was measured for 10 seconds with a VictorX3 multimodal plate reader (Perkin Elmer).

### Luminescent multiplex viability assay

The luminescent multiplex viability assay (LMVA) measures first the RLuc activity in live cells with EnduRen (Promega) and next measures the cell ATP content with the luminescent CellTiter-Glo reagent (Promega) within the same assay wells. Luminescence in CPS was measured with a VictorX3 multimodal plate reader (Perkin Elmer). The EnduRen reagent was used according to the manufacturer’s instructions (Promega). Forty-five μl of trypanosome suspension was transferred to a half area white opaque 96 well plate (Perkin Elmer) to which 5 μl of 60 μM EnduRen in HH (or 5 μl of HH) was added. After 2 hours incubation at 37° C and a 10 minutes equilibration at 25°C, the luminescence was measured by integrating the number of photons per 1 second. Then, an equal volume (50 μl) of reconstituted CellTiter-Glo reagent (Promega) was added to this parasite suspension, after 2 minutes of shaking, and a 10 minutes delay, the number of photons per second was integrated.

### LMVA performance

To assess the lower detection limit of wild type and recombinant trypanosome cells in HH, log phase trypanosomes at 10^5^ cells ml^-1^ were harvested, concentrated and a tenfold dilution series was made in triplicate from 10^6^ down to 10^2^ cells ml^-1^ in 100 μl. This series was tested with the LMVA (using a 45 μl trypanosome suspension, as described above) and the RLAS (using 40 μl, as described above). The luminescent values (in CPS) of the cell containing samples were divided by the value of the blanks without cells (signal to background) and this relative luminescence value was plotted against the number of trypanosomes for each assay. Linear fits were used to find the lower detection limit of the number of trypanosome cells in each assay (at a signal to background ratio of 3 to 1). To measure the performance of the LMVA during the whole growth period, a trypanosome suspension of 2 × 10^4^ cells ml^-1^ in 100 μl was inoculated in 15 wells and every day, for four days, triplicate wells were sampled for cell counting (using 20 μl cell suspension, as described above) and for the LMVA (using 45 μl cell suspension, as described above). Doubling times were calculated using non-linear regression in Prism (Graphpad).

### Drug sensitivity testing

Eflornithine (Sanofi Aventis) and hygromycin B (Sigma) were prepared as 10 mg ml^-1^ stock solutions in distilled water. Melarsoprol (Arsobal, Sanofi Aventis), pentamidine isethionate (Sanofi Aventis) and nifurtimox (Sigma) were stored as 10 mg ml^-1^ stock solutions in DMSO. A method to measure the IC_50_ values of compounds in 96-well plates was performed as described elsewhere [48]. Threefold drug dilutions in triplicate were made in HH from 100 to 0.14 μg ml^-1^ for eflornithine and hygromycin, from 50 to 0.07 μg ml^-1^ for nifurtimox and from 500 to 0.7 ng ml^-1^ for pentamidine and melarsoprol. Each drug concentration was inoculated with either 2 × 10^4^ cells ml^-1^ or 5 × 10^3^ cells ml^-1^ in a final volume of 100 μl. Next the plate was incubated for 72 hours. For detection of hygromycin sensitivity in the fluorimetric resazurin assay, 10 μl of 12 mg resazurin in 100 ml PBS were added to 100 μl trypanosome suspension in a 96 well transparent plate (Nunc). Alternatively, for comparison of IC_50_ values between the LMVA and resazurin assay, the 100 μl trypanosome suspension was split: 45 μl were used for LMVA as described above and 45 μl were transferred to a half area black opaque plate (Perkin Elmer) containing 5 μl of resazurin. After 24 h at 37°C, fluorescence was measured (excitation λ = 560 nm; emission λ = 590 nm) with a VictorX3 multimodal plate reader using top reading (Perkin Elmer) [26]. The results were expressed as the percent reduction in parasite viability compared to parasite viability in control wells without drugs, and a 50% inhibitory concentration (IC_50_) was calculated using non-linear regression in Prism (GraphPad).

## Results

### *In vitro* adaptation in HH

**Figure 1:**
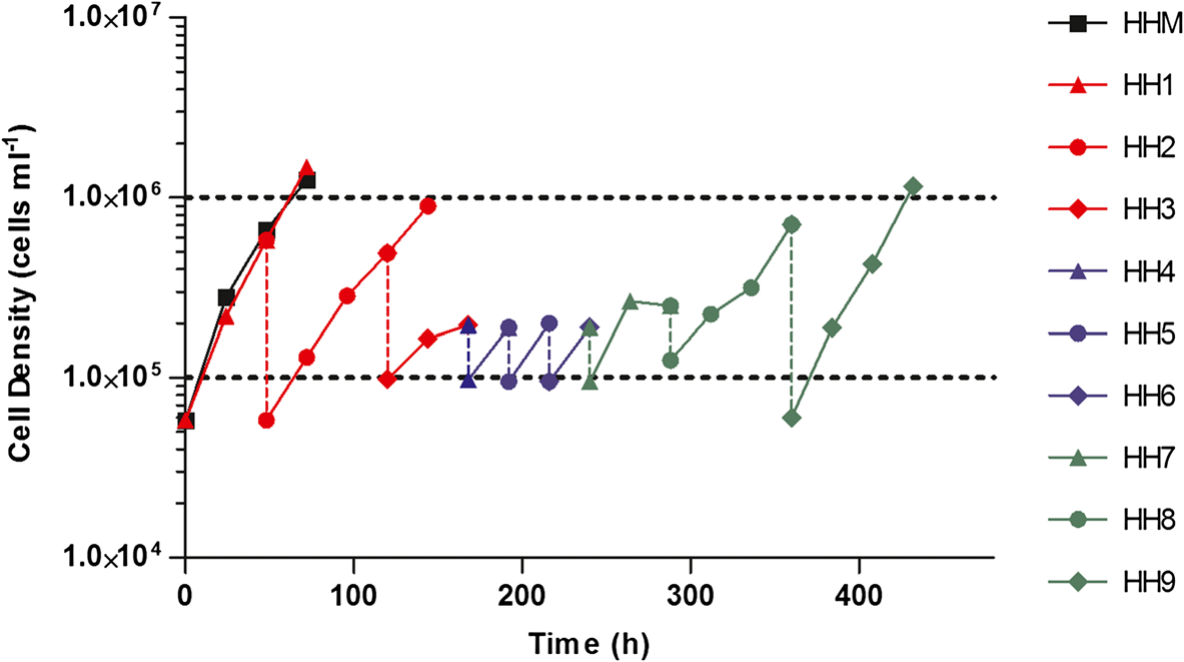
**Growth profiles of an HHM line, and from the nine subpassages (HH1 – HH9) that resulted in the HH adapted line of *T.b. gambiense *348 BT.** Vertical dotted lines mark the start of each new subpassage made using 90 to 50% new medium.

To be compatible with compound screening, it was necessary to adapt the *T.b. gambiense* 348BT strain that readily grows in HHM to a medium without methylcellulose because the latter is very viscous, which renders homogenous volume distribution and centrifugation very difficult. The strain was adapted to HH medium by gradually diluting out the initial 1.1% (w/v) methylcellulose concentration in the HHM. With each daily subpassage, 1 to 5 volumes of the trypanosome suspension was inoculated in 9 to 5 volumes of HH, making sure that the starting cell concentration was between 5 × 10^4^ and 1 × 10^5^ cells ml^-1^. In many instances this approach was not successful and most subpassages resulted in cell culture cessation. One population survived eight subpassages in HH, whereafter its growth profile became similar to the original profile in HHM. When inoculated at ± 10^5^ cells ml^-1^, the logarithmic growth phase lasted 72 hours with a maximum population density of ± 10^6^ cells ml^-1^. This population was considered fully adapted to HH and used as such in further experiments. Figure 1 shows the growth profiles of the HHM adapted line, and from nine subpassages (HH1 – HH9) that resulted in this HH adapted line.

### Transfection with pHD309 RLuc

We transfected the HH adapted line of *T.b. gambiense* 348 BT with plasmid pHD309 RLuc and obtained 3 recombinant populations from 2 independent transfections: population #2.1, #3.1 and #3.2. Hygromycin resistant populations were evident after 8 days of selection. After 4 weeks in 1 μg ml^-1^ hygromycin, the IC_50_ value for hygromycin, starting with a cell density of 2 × 10^4^ cells ml^-1^, was calculated using the fluorimetric resazurin assay for wild type and recombinant populations. All recombinant populations were at least twenty times more resistant to hygromycin than the wild type population (Table 1).

**Table 1:** **Sensitivity to hygromycin and lower detection limits of RLAS, EnduRen and CellTiter-Glo with the *T.b. gambiense *348BT wild type and three RLuc transfected strains**

**Population**	**IC **_**50 **_**hygromycin**^**a**^	**RLAS**^**b**^	**EnduRen**^**c**^	**CellTiter-Glo**^**d**^
wild type	< 0.12	n.a.	n.a.	218 ± 14
RLuc #2.1	7.5 ± 1.1	207 ± 22	991 ± 131	212 ± 9
RLuc #3.1	2.4 ± 0.7	810 ± 80	4804 ± 511	221 ±16
RLuc #3.2	4.2 ± 0.9	488 ± 50	3225 ± 423	215 ± 22

^a^ reported in μg ml^-1^ ± SD, from 3 cultures and determined with fluorimetric assay for measurement of reduction of resazurin.

^b^ reported in number of cells ± SD, from 3 cultures and determined with RLAS, a luminescent assay for detection of *Renilla* luciferase activity in lysed cells.

^c^ reported in number of cells ± SD, from 3 cultures and determined with EnduRen, a luminescent assay for detection of *Renilla* luciferase activity in living cells.

^d^ reported in number of cells ± SD, from 3 cultures and determined with CellTiter-Glo, a luminescent assay for quantification of ATP.

n.a. = not applicable.

### Activity of RLuc

To select the most luminescent population, activity of the expressed luciferase in the wild type and recombinant populations was quantified with two assays that measure RLuc activity either in lysed cells (RLAS, Promega) or in live cells (EnduRen, Promega). With both RLuc activity assays, a linear fit between the number of log phase recombinant trypanosomes and relative luminescence (signal to background) data was obtained until up to 10^6^ cells ml^-1^, the most dense trypanosome suspension tested. The lower detection limit generated from these linear fits was different for each of the recombinant populations and was also different between both RLuc assays (ANOVA, *p* < 0.05) (Table 1). Due to its lowest detection limit in both RLuc assays, population #2.1 was identified as the most luminescent population.

### LMVA performed on cells in the logarithmic growth phase

**Figure 2:**
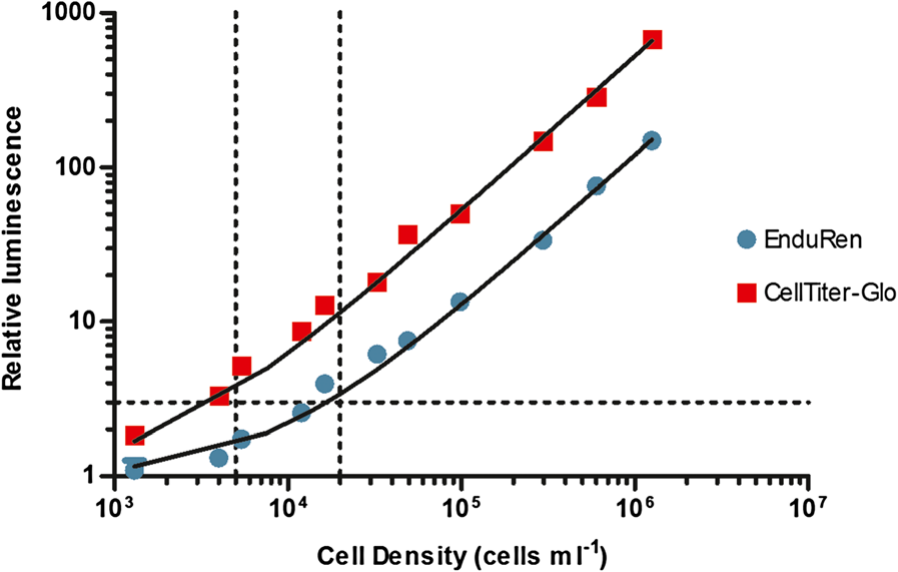
**Relative luminescence of LMVA in function of cell density tested with population RLuc # 2.1.** Horizontal dotted line represents a signal to background ratio of 3 to 1.Vertical dotted lines mark the cell density necessary for detection at this signal to background ratio (5 × 10^3^ cells ml^-1^ for CellTiter-Glo and 2 × 10^4^ cells ml^-1^ for EnduRen).

The CellTiter-Glo assay was used to measure the luminescence of the ATP content of wild type and recombinant populations. A linear fit between the number of trypanosomes and relative luminescence signal could be found up to 10^6^ cells ml^-1^, the most dense trypanosome suspension tested. The lower detection limits generated from these linear fits were equal for wild type and each recombinant population (ANOVA, *p* > 0.05) (Table 1). Importantly, the detection limit in the CellTiter-Glo assay was not altered, whether or not the EnduRen substrate was included in the medium (ANOVA, *p* > 0.05). This allows the EnduRen assay to be combined with the CellTiter-Glo assay in one multiplexed luminescent format, i.e. the LMVA, reporting thus both on the signal from the RLuc expression as well as on the signal of the ATP viability assay from the same wells. Figure 2 shows that for the most luminescent population, RLuc #2.1, we require at least ± 2 × 10^4^ cells ml^-1^ to obtain a signal to background ratio of at least 3 to 1 for the EnduRen assay, while the CellTiter-Glo assay requires only ± 5 × 10^3^ cells ml^-1^.

### LMVA performance during growth profile

**Figure 3:**
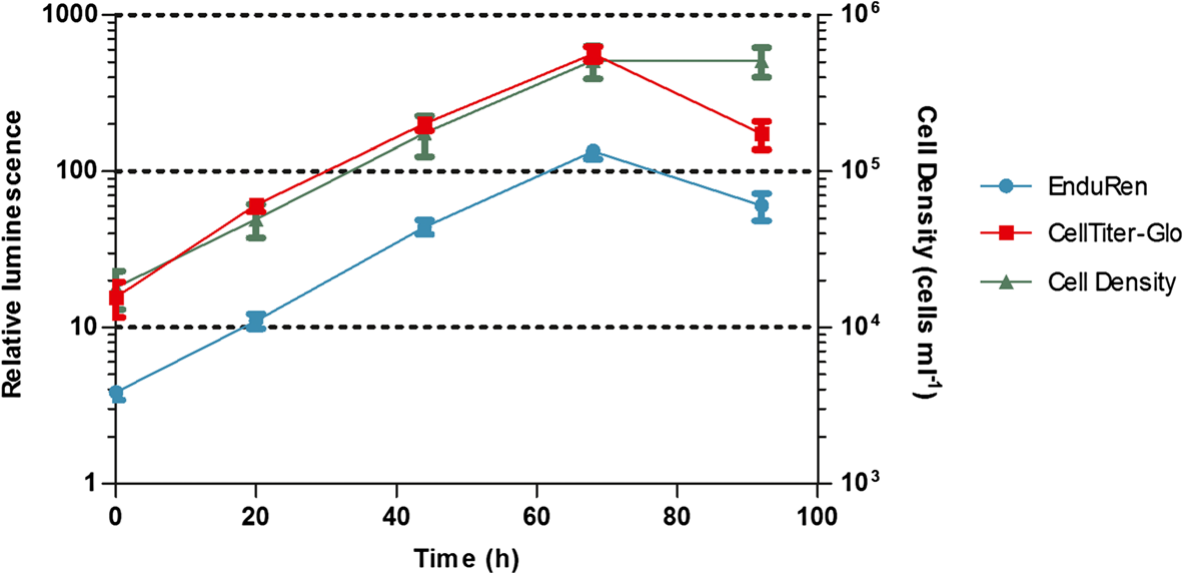
Growth curve of RLuc# 2.1 in function of time, assessed by LMVA (in relative luminescence, left Y-axis) and by microscopy (cell density, right Y-axis).

The growth profiles of *T.b. gambiense* 348BT wild type and RLuc #2.1 were established by the LMVA and by daily cell counting during a 5 time-points experiment lasting 96 hours. Figure 3 shows that at a starting concentration of 2 × 10^4^ cells ml^-1^, both arms of the LMVA could record the exponential growth of the RLuc population during the first 72 hours. After 96 hours, when the population was in stationary phase, the output of both luminescent assays was lower than expected from the cell density. Doubling times in the exponential growth phase calculated with LMVA data, using either EnduRen or CellTiter-Glo, did not differ significantly from the doubling time calculated with the cell counting data nor did these growth rates differ between wild type and RLuc #2.1 (ANOVA, *p* > 0.05) (Table 2).

**Table 2:** ***In vitro *doubling time of the *T.b. gambiense *348BT wild type and the recombinant RLuc #2.1 strain assessed in triplicate with EnduRen, CellTiter-Glo and by microscopy**

**Population**	**Doubling time**^**a**^		
	**EnduRen**^**b**^	**CellTiter-Glo**^**c**^	**Microscopy**
wild type	n.a.	12.7 ± 0.8	12.6 ± 0.9
RLuc #2.1	12.9 ± 0.7	12.4 ± 0.7	12.6 ± 0.5

^a^ reported in h^-1^ ± SD, from 3 cultures.

^b^ luminescent assay for measurement of *Renilla* luciferase activity in living cells.

^c^ luminescent assay for quantification of ATP.

n.a. not applicable.

### LMVA performance in drug sensitivity screening

The IC_50_ values for pentamidine, eflornithine, melarsoprol and nifurtimox were compared between the wild type and the RLuc #2.1 populations, between one or both arms of the LMVA and the fluorimetric resazurin viability assay and between using 2 × 10^4^ or 5 × 10^3^ cells ml^-1^ as starting concentration (Table 3). We could not observe a significant difference in IC_50_ values between the wild type and the RLuc population for any given viability assay (ANOVA, *p* > 0.05) or starting concentration (ANOVA, *p >* 0.05) when using the same drug. Furthermore, for eflornithine, melarsoprol and nifurtimox, there was no variation in IC_50_ values obtained from the different viability assays (ANOVA, *p* > 0.05) when using the same starting concentration. However, for these drugs, all viability assays recorded significant differences in IC_50_ values between using 2 × 10^4^ or 5 × 10^3^ cells ml^-1^ as a starting concentration (ANOVA, *p* < 0.05). In contrast, for pentamidine, we observed higher IC_50_ values in the resazurin assay than in either the EnduRen or CellTiter-Glo assay (ANOVA, *p* < 0.05) when using the same starting concentration. However, the differences in IC_50_ values between using 2 × 10^4^ and 5 × 10^3^ cells ml^-1^ as a starting concentration were not significant for this drug (ANOVA, *p* > 0.05). We conclude that the presence of pHD 309 *RLuc* does not influence the susceptibility of *T.b. gambiense* 348 BT to any of the tested drugs, but a particular drug may influence the IC_50_ value when tested with a different viability assay (as for pentamidine), or when a different starting concentration is used (for eflornithine, nifurtimox and melarsoprol).

**Table 3:** **IC**_**50 **_**values for eflornithine, melarsoprol, pentamidine and nifurtimox obtained with the *T.b. gambiense *348BT wild type and the recombinant RLuc #2.1 strain assessed with EnduRen, CellTiter-Glo and resazurin**

**Inoculum**	**Drug**	**IC**_**50**_^**a**^					
		**EnduRen**^**d**^		**CellTiter-Glo**^**e**^		**Resazurin**^**f**^	
		**wild type**	**RLuc # 2.1**	**wild type**	**RLuc # 2.1**	**wildtype**	**RLuc # 2.1**
5 x 10^3^	eflornithine^b^	n.a.	1.0 ± 0.4	1.1 ± 0.4	1.0 ± 0.5	1.1 ± 0.6	1.4 ± 0.4
	melarsoprol^c^	n.a.	5.5 ± 2.1	4.5 ± 2.2	5.0 ± 1.9	6.3 ± 3.0	6.5 ± 2.3
	pentamidine^c^	n.a.	40.1 ± 11.1	43.6 ± 10.3	41.1 ± 7.5	67.5 ± 11.1	64.1 ± 13.6
	nifurtimox^c^	n.a.	334 ± 47	274 ± 93	380 ± 84	437 ± 139	410 ± 132
2 x 10^4^	eflornithine^b^	n.a.	2.8 ± 0.5	3.0 ± 0.7	2.6 ± 1.0	2.6 ± 0.9	2.9 ± 0.7
	melarsoprol^c^	n.a.	11.0 ± 3.2	12.0 ± 2.8	11.5 ± 2.6	8.9 ± 2.2	11.9 ± 2.6
	pentamidine^c^	n.a.	47.5 ± 8.1	43.7 ± 10.8	48.9 ± 9.1	74.7 ± 12.3	72.5 ± 6.3
	nifurtimox^c^	n.a.	700 ± 63	670 ± 78	720 ± 75	751 ± 174	768 ± 125

^a^ values are the mean ± SD from 4 cultures.

^b^ reported in μg ml^-1^.

^c^ reported in ng ml^-1^.

^d^ luminescent assay for measurement of *Renilla* luciferase activity in living cells.

^e^ luminescent assay for quantification of ATP.

^f^ fluorimetric assay for measurement of reduction of resazurin.

n.a. not applicable.

### *In vivo* infections of wild type and recombinant 348BT

Differences in infectivity, as determined by the number of days until the first parasite is detected (prepatent period), and virulence, as determined by the number of days of survival of the rodents, between the original cell line (adapted *in vivo* but not *in vitro*), the HHM and HH (adapted *in vitro*) cell lines, and the resulting RLuc cell line, were compared by expanding each population in 4 mice treated or not with endoxan. All mice were found parasitemic after 3 to 7 days of infection. The mean prepatent period was not significantly different between mice treated or not with endoxan and between the different trypanosome cell lines (data not shown). During the next 3 weeks of follow up, we could sporadically detect waves of parasitemia in all mice of all groups. No mice died from the infection during the experiment and all mice were sacrificed at day 30.

## Discussion

This study was undertaken to develop a *Renilla* luciferase based luminescent multiplex viability assay (LMVA) for *in vitro* compound screening on bloodstream form *T.b. gambiense*. To obtain the *RLuc* transfected *T.b. gambiens*e, we started with a recently isolated strain that underwent few *in vivo* passages in rodents and that was adapted to *in vitro* HMI-9 based medium with human serum (HH). Although nucleofection has been described to be very efficient for transient and for stable transfection of African trypanosomes [29,49,50], our study confirms that transfection with pHD309 *RLuc* is also successful by electroporation [40]. Due to the presence of hygromycin phosphotransferase as resistance selection marker for stable genomic integration of *Renilla* luciferase, cross resistance against trypanotoxic hygromycin analogues may pose a problem, as has been described for pyrimethamine resistance of a transgenic firefly luciferase *Plasmodium* strain [34]. To select the most RLuc transfected trypanosome population, two RLuc activity assays were used. The RLAS system is very sensitive, but is not compatible with CellTiter-Glo and requires more manipulations than the EnduRen assay. In the EnduRen assay, only the most hygromycin resistant population allowed fast detection of low numbers of cells (± 2 × 10^4^ cells ml^-1^). In contrast to the EnduRen assay, the CellTiter-Glo assay does not require genetic manipulation of the trypanosome strain, the assay is performed faster and has a lower detection limit (± 5 × 10^3^ cells ml^-1^). Combining the EnduRen assay with CellTiter-Glo as two independent assays measuring respectively the RLuc activity and the ATP content of the cells, we were able to establish a multiplex viability assay for which the luminescence signals correlate well with the cell density during the proliferation of the long slender bloodstream form trypanosomes. The multiplex luminescent format has several advantages over the fluorimetric resazurin assay: first, viability assessment requires less incubation time with substrate before detection takes place (2 hours vs 16 – 24 hours) and second, two independent viability parameters are assayed in the same experiment (RLuc activity and ATP content). Disadvantages of this LMVA are its higher cost compared to the resazurin assay and the need for an *RLuc* transfected trypanosome strain*.* On the other hand, transfecting a trypanosome strain with a luminescence reporter gene makes it possible to first select trypanocidal compounds *in vitro* and subsequently test their activity *in vivo* with the same trypanosome strain by bioluminescence imaging of infected mice. Here we confirm that even after transfection with pHD 309 *RLuc*, the *in vitro* and *in vivo* growth characteristics of the recombinant *T.b. gambiense* strain are not different from the wild type strain. We used several drugs that are in use against *T.b. gambiense* sleeping sickness to investigate whether the pHD 309 *RLuc* integration would have altered the drug sensitivity profile compared to the wild type. No such influence could be observed. Furthermore, the IC_50_ values for these drugs fall within range of other *T.b. gambiense* field strains isolated in Mbuji-Mayi, Democratic Republic of the Congo, that have been used recently for validation of fexinidazole [20,26]. Before the present LMVA can be adopted as HTS assay, further evaluation and optimisation is necessary. Also, the assay should be tested for its applicability on other RLuc transfected *Trypanozoon* strains that have become available recently, including *T.b. brucei*, *T.b. rhodesiense* and *T. evansi* strains.

## Conclusions

In conclusion, we showed that a luminescent multiplex viability assay with an *RLuc* transfected *T.b. gambiense* strain can be used as an alternative to the resazurin viability assay in drug discovery. Both the LMVA assay and the trypanosome strain represent valuable assets in the fight against sleeping sickness, complementing the available tools for HTS compound screening, particularly where it comes to confirm *in vivo* trypanocidal activity of molecules that have been selected *in vitro*.

## Competing interests

The authors declare that they have no competing interests.

## Authors’ contributions

NVR carried out the adaptation of the strain *in vitro*, did the transfection and selection of the luminescent clones, performed the luminescent and fluorescent activity assays, did the statistical analysis and drafted the manuscript. PP isolated and adapted the strain *in vivo.* SR revised the manuscript. FC made the luminescent vector pHD309 RLuc and obtained funding for this manuscript and PB participated in the design of the study, obtained funding and did the critical revision of the manuscript. All authors read and approved the final version of the manuscript.

## References

[B1] Simarro PP Diarra A Ruiz Postigo JA Franco JR Jannin JG The human african trypanosomiasis control and surveillance programme of the world health organization 2000–2009: the way forward PLoS Negl Trop Dis 2011 5 e1007 10.1371/journal.pntd.0001007 21364972 PMC3042999

[B2] Masocha W Kristensson K Passage of parasites across the blood–brain barrier Virulence 2012 3 202 212 22460639 10.4161/viru.19178 PMC3396699

[B3] Steverding D The development of drugs for treatment of sleeping sickness: a historical review Parasit Vectors 2010 3 15 10.1186/1756-3305-3-15 20219092 PMC2848007

[B4] Mäser P Wittlin S Rottmann M Wenzler T Kaiser M Brun R Antiparasitic agents: new drugs on the horizon Curr Opin Pharmacol 2012 12 562 566 10.1016/j.coph.2012.05.001 22652215

[B5] Mackey ZB Koupparis K Nishino M McKerrow JH High-throughput analysis of an RNAi library identifies novel kinase targets in *Trypanosoma brucei* Chem Biol Drug Des 2011 78 454 463 10.1111/j.1747-0285.2011.01156.x 21668652 PMC3166884

[B6] Mesia GK Tona GL Nanga TH Cimanga RK Apers S Cos P Maes L Pieters L Vlietinck AJ Antiprotozoal and cytotoxic screening of 45 plant extracts from Democratic Republic of Congo J Ethnopharmacol 2008 115 409 415 10.1016/j.jep.2007.10.028 18068320

[B7] Sykes ML Avery VM A luciferase based viability assay for ATP detection in 384-well format for high throughput whole cell screening of *Trypanosoma brucei brucei* bloodstream form strain 427 Parasit Vectors 2009 2 54 10.1186/1756-3305-2-54 19909542 PMC2781010

[B8] Sykes ML Avery VM Development of an Alamar Blue viability assay in 384-well format for high throughput whole cell screening of *Trypanosoma brucei brucei* bloodstream form strain 427 AmJTrop Med Hyg 2009 81 665 674 10.4269/ajtmh.2009.09-0015 19815884

[B9] Jones DC Hallyburton I Stojanovski L Read KD Frearson JA Fairlamb AH Identification of a kappa-opioid agonist as a potent and selective lead for drug development against human African trypanosomiasis Biochem Pharmacol 2010 80 1478 1486 10.1016/j.bcp.2010.07.038 20696141 PMC3025325

[B10] Ang KK Ratnam J Gut J Legac J Hansell E Mackey ZB Skrzypczynska KM Debnath A Engel JC Rosenthal PJ Mining a cathepsin inhibitor library for new antiparasitic drug leads PLoS Negl Trop Dis 2011 5 e1023 10.1371/journal.pntd.0001023 21572521 PMC3086806

[B11] Brand S Cleghorn LA McElroy SP Robinson DA Smith VC Hallyburton I Harrison JR Norcross NR Spinks D Bayliss T Discovery of a novel class of orally active trypanocidal N-myristoyltransferase inhibitors J Med Chem 2012 55 140 152 10.1021/jm201091t 22148754 PMC3256935

[B12] Navarro G Chokpaiboon S De MG Bray WM Nisam SC McKerrow JH Pudhom K Linington RG Hit-to-lead development of the chamigrane endoperoxide merulin A for the treatment of African sleeping sickness PLoS One 2012 7 e46172 10.1371/journal.pone.0046172 23029428 PMC3459870

[B13] Bowling T Mercer L Don R Jacobs R Nare B Application of a resazurin-based high-throughput screening assay for the identification and progression of new treatments for human African trypanosomiasis Int J Parasitol Drugs Drug Resist 2012 2 262 270 24533287 10.1016/j.ijpddr.2012.02.002 PMC3862424

[B14] De Rycker M O’Neill S Joshi D Campbell L Gray DW Fairlamb AH A static-cidal assay for *Trypanosoma brucei* to aid hit prioritisation for progression into drug discovery programmes PLoS Negl Trop Dis 2012 6 e1932 10.1371/journal.pntd.0001932 23209868 PMC3510075

[B15] Kaminsky R Brun R *In vitro* and *in vivo* activities of trybizine hydrochloride against various pathogenic trypanosome species Antimicrob Agents Chemother 1998 42 2858 2862 9797216 10.1128/aac.42.11.2858 PMC105956

[B16] Wenzler T Boykin DW Ismail MA Hall JE Tidwell RR Brun R New treatment option for second-stage African sleeping sickness: in vitro and in vivo efficacy of aza analogs of DB289 Antimicrob Agents Chemother 2009 53 4185 4192 10.1128/AAC.00225-09 19620327 PMC2764217

[B17] Nare B Wring S Bacchi C Beaudet B Bowling T Brun R Chen D Ding C Freund Y Gaukel E Discovery of novel orally bioavailable oxaborole 6-carboxamides that demonstrate cure in a murine model of late-stage central nervous system African trypanosomiasis Antimicrob Agents Chemother 2010 54 4379 4388 10.1128/AAC.00498-10 20660666 PMC2944573

[B18] Torreele E Bourdin TB Tweats D Kaiser M Brun R Mazue G Bray MA Pecoul B Fexinidazole - a new oral nitroimidazole drug candidate entering clinical development for the treatment of sleeping sickness PLoS Negl Trop Dis 2010 4 e923 10.1371/journal.pntd.0000923 21200426 PMC3006138

[B19] Jacobs RT Nare B Wring SA Orr MD Chen D Sligar JM Jenks MX Noe RA Bowling TS Mercer LT SCYX-7158, an Orally-Active Benzoxaborole for the Treatment of Stage 2 Human African Trypanosomiasis PLoS Negl Trop Dis 2011 5 e1151 10.1371/journal.pntd.0001151 21738803 PMC3125149

[B20] Kaiser M Bray MA Cal M Bourdin TB Torreele E Brun R Antitrypanosomal activity of fexinidazole, a new oral nitroimidazole drug candidate for treatment of sleeping sickness Antimicrob Agents Chemother 2011 55 5602 5608 10.1128/AAC.00246-11 21911566 PMC3232772

[B21] Bacchi CJ Nathan HC Livingston T Valladares G Saric M Sayer PD Njogu AR Clarkson AB Jr Differential susceptibility to DL-alpha-difluoromethylornithine in clinical isolates of *Trypanosoma brucei rhodesiense* Antimicrob Agents Chemother 1990 34 1183 1188 10.1128/AAC.34.6.1183 2118325 PMC171781

[B22] Maina NW Oberle M Otieno C Kunz C Maeser P Ndung’u JM Brun R Isolation and propagation of *Trypanosoma brucei gambiense* from sleeping sickness patients in south Sudan Trans R Soc Trop Med Hyg 2007 101 540 546 10.1016/j.trstmh.2006.11.008 17275053

[B23] Pyana PP Ngay Lukusa I Mumba Ngoyi D Van Reet N Kaiser M Karhemere Bin Shamamba S Büscher P Isolation of *Trypanosoma brucei gambiense* from cured and relapsed sleeping sickness patients and adaptation to laboratory mice PLoS Negl Trop Dis 2011 5 e1025 10.1371/journal.pntd.0001025 21526217 PMC3079580

[B24] Jackson AP Sanders M Berry A McQuillan J Aslett MA Quail MA Chukualim B Capewell P MacLeod A Melville SE The genome sequence of *Trypanosoma brucei gambiense*, causative agent of chronic human African trypanosomiasis PLoS Negl Trop Dis 2010 4 e658 10.1371/journal.pntd.0000658 20404998 PMC2854126

[B25] Iten M Matovu E Brun R Kaminsky R Innate lack of susceptibility of Ugandan *Trypanosoma brucei rhodesiense* to DL-alfa-difluoromethylornithine (DFMO) Trop Med Parasitol 1995 46 190 194 8533023

[B26] Raz B Iten M Grether-Buhler Y Kaminsky R Brun R The Alamar Blue assay to determine drug sensitivity of African trypanosomes (*T.b. rhodesiense* and *T.b. gambiense*) in vitro Acta Trop 1997 68 139 147 10.1016/S0001-706X(97)00079-X 9386789

[B27] Bacchi CJ Chemotherapy of human african trypanosomiasis Interdiscip Perspect Infect Dis 2009 2009 195040 19707529 10.1155/2009/195040 PMC2730475

[B28] Van Reet N Pyana PP Deborggraeve S Büscher P Claes F *Trypanosoma brucei gambiense*: HMI-9 medium containing methylcellulose and human serum supports the continuous axenic in vitro propagation of the bloodstream form Exp Parasitol 2011 128 285 290 10.1016/j.exppara.2011.02.018 21354143

[B29] Giroud C Ottones F Coustou V Dacheux D Biteau N Miezan B Van Reet N Carrington M Doua F Baltz T Murine models for *Trypanosoma brucei gambiense* disease progression-from silent to chronic infections and early brain tropism PLoS Negl Trop Dis 2009 3 e509 10.1371/journal.pntd.0000509 19721701 PMC2728506

[B30] Brun R Baeriswyl S Kunz C In vitro drug sensitivity of *Trypanosoma gambiense* isolates Acta Trop 1989 46 369 376 10.1016/0001-706X(89)90049-1 2575872

[B31] Gould MK Vu XL Seebeck T de Koning HP Propidium iodide-based methods for monitoring drug action in the *Kinetoplastidae*: comparison with the Alamar Blue assay Anal Biochem 2008 382 87 93 10.1016/j.ab.2008.07.036 18722997

[B32] Merschjohann K Sporer F Steverding D Wink M In vitro effect of alkaloids on bloodstream forms of *Trypanosoma brucei* and *T. congolense* Planta Med 2001 67 623 627 10.1055/s-2001-17351 11582539

[B33] Cui L Miao J Wang J Li Q Cui L *Plasmodium falciparum*: development of a transgenic line for screening antimalarials using firefly luciferase as the reporter Exp Parasitol 2008 120 80 87 10.1016/j.exppara.2008.05.003 18579134 PMC2559859

[B34] Che P Cui L Kutsch O Cui L Li Q Validating a firefly luciferase-based high-throughput screening assay for antimalarial drug discovery Assay Drug Dev Technol 2012 10 61 68 10.1089/adt.2011.0378 22050430 PMC3277734

[B35] Sereno D Roy G Lemesre JL Papadopoulou B Ouellette M DNA transformation of Leishmania infantum axenic amastigotes and their use in drug screening Antimicrob Agents Chemother 2001 45 1168 1173 10.1128/AAC.45.4.1168-1173.2001 11257031 PMC90440

[B36] Michel G Ferrua B Lang T Maddugoda MP Munro P Pomares C Lemichez E Marty P Luciferase-expressing *Leishmania infantum* allows the monitoring of amastigote population size, in vivo, ex vivo and in vitro PLoS Negl Trop Dis 2011 5 e1323 10.1371/journal.pntd.0001323 21931877 PMC3172198

[B37] Pulido SA Munoz DL Restrepo AM Mesa CV Alzate JF Velez ID Robledo SM Improvement of the green fluorescent protein reporter system in *Leishmania spp.* for the in vitro and in vivo screening of antileishmanial drugs Acta Trop 2012 122 36 45 10.1016/j.actatropica.2011.11.015 22155571

[B38] Bot C Hall BS Bashir N Taylor MC Helsby NA Wilkinson SR Trypanocidal activity of aziridinyl nitrobenzamide prodrugs Antimicrob Agents Chemother 2010 54 4246 4252 10.1128/AAC.00800-10 20679506 PMC2944584

[B39] Canavaci AM Bustamante JM Padilla AM Perez Brandan CM Simpson LJ Xu D Boehlke CL Tarleton RL In vitro and in vivo high-throughput assays for the testing of anti-*Trypanosoma cruzi* compounds PLoS Negl Trop Dis 2010 4 e740 10.1371/journal.pntd.0000740 20644616 PMC2903469

[B40] Claes F Vodnala SK Van Reet N Boucher N Lunden-Miguel H Baltz T Goddeeris BM Büscher P Rottenberg ME Bioluminescent imaging of *Trypanosoma brucei* shows preferential testis dissemination which may hamper drug efficacy in sleeping sickness patients PLoS Negl Trop Dis 2009 3 e486 10.1371/journal.pntd.0000486 19621071 PMC2707598

[B41] Vodnala SK Ferella M Lunden-Miguel H Betha E Van Reet N Amin DN Oberg B Andersson B Kristensson K Wigzell H Preclinical assessment of the treatment of second-stage African trypanosomiasis with cordycepin and deoxycoformycin PLoS Negl Trop Dis 2009 3 e495 10.1371/journal.pntd.0000495 19652702 PMC2713411

[B42] Xie X Wang QY Xu HY Qing M Kramer L Yuan Z Shi PY Inhibition of dengue virus by targeting viral NS4B protein J Virol 2011 85 11183 11195 10.1128/JVI.05468-11 21865382 PMC3194949

[B43] Gilbert DF Erdmann G Zhang X Fritzsche A Demir K Jaedicke A Muehlenberg K Wanker EE Boutros M A novel multiplex cell viability assay for high-throughput RNAi screening PLoS One 2011 6 e28338 10.1371/journal.pone.0028338 22162763 PMC3230607

[B44] McCulloch R Vassella E Burton P Boshart M Barry JD Transformation of monomorphic and pleomorphic *Trypanosoma brucei* Methods Mol Biol 2004 262 53 86 14769956 10.1385/1-59259-761-0:053

[B45] Mumba Ngoyi D Lejon V Pyana P Boelaert M Ilunga M Menten J Mulunda JP Van Nieuwenhove S Muyembe Tamfum JJ Büscher P How to shorten patient follow-up after treatment for *Trypanosoma brucei gambiense* sleeping sickness? J Infect Dis 2010 201 453 463 10.1086/649917 20047500

[B46] Lanham SM Godfrey DG Isolation of salivarian trypanosomes from man and other mammals using DEAE-cellulose Exp Parasitol 1970 28 521 534 10.1016/0014-4894(70)90120-7 4993889

[B47] Herbert WJ Lumsden WHR *Trypanosoma brucei:* A rapid “matching” method for estimating the host’s parasitemia Exp Parasitol 1976 40 427 431 10.1016/0014-4894(76)90110-7 976425

[B48] Gillingwater K Büscher P Brun R Establishment of a panel of reference *Trypanosoma evansi* and *Trypanosoma equiperdum* strains for drug screening Vet Parasitol 2007 148 114 121 10.1016/j.vetpar.2007.05.020 17624671

[B49] Burkard G Fragoso CM Roditi I Highly efficient stable transformation of bloodstream forms of *Trypanosoma brucei* Mol Biochem Parasitol 2007 153 220 223 10.1016/j.molbiopara.2007.02.008 17408766

[B50] Coustou V Guegan F Plazolles N Baltz T Complete in vitro life cycle of *Trypanosoma congolense*: development of genetic tools PLoS Negl Trop Dis 2010 4 e618 10.1371/journal.pntd.0000618 20209144 PMC2830455

